# SGI: automatic clinical subgroup identification in omics datasets

**DOI:** 10.1093/bioinformatics/btab656

**Published:** 2021-09-16

**Authors:** Mustafa Buyukozkan, Karsten Suhre, Jan Krumsiek

**Affiliations:** Department of Physiology and Biophysics, Institute for Computational Biomedicine, New York, NY 10021, USA; Englander Institute for Precision Medicine, Weill Cornell Medicine, New York, NY 10021, USA; Englander Institute for Precision Medicine, Weill Cornell Medicine, New York, NY 10021, USA; Department of Physiology and Biophysics, Weill Cornell Medicine-Qatar, Education City, 24144 Doha, Qatar; Department of Physiology and Biophysics, Institute for Computational Biomedicine, New York, NY 10021, USA; Englander Institute for Precision Medicine, Weill Cornell Medicine, New York, NY 10021, USA

## Abstract

**Summary:**

The ‘Subgroup Identification’ (SGI) toolbox provides an algorithm to automatically detect clinical subgroups of samples in large-scale omics datasets. It is based on hierarchical clustering trees in combination with a specifically designed association testing and visualization framework that can process an arbitrary number of clinical parameters and outcomes in a systematic fashion. A multi-block extension allows for the simultaneous use of multiple omics datasets on the same samples. In this article, we first describe the functionality of the toolbox and then demonstrate its capabilities through application examples on a type 2 diabetes metabolomics study as well as two copy number variation datasets from The Cancer Genome Atlas.

**Availability and implementation:**

SGI is an open-source package implemented in R. Package source codes and hands-on tutorials are available at https://github.com/krumsieklab/sgi. The QMdiab metabolomics data is included in the package and can be downloaded from https://doi.org/10.6084/m9.figshare.5904022.

**Supplementary information:**

Supplementary data are available at *Bioinformatics* online.

## 1 Introduction

The identification of patient subgroups from high-dimensional molecular profiles has become a central approach in biomedical research, driven by the wide availability of modern ‘multi-omics’ datasets (Collisson *et al.*, 2011; Eddy *et al.*, 2020; Rouzier *et al.*, 2005). The central idea is that genomics, transcriptomics, proteomics, metabolomics and other deep molecular phenotypes will inherently define groups of patients that are similar with respect to disease-relevant clinical outcomes. Note that, ‘outcome’ is here defined in a statistical sense, and includes parameters such as sex, prevalent disease and current BMI. Recent examples of molecularly defined subgroups include the identification of subtypes of lymphoma that severely impact survival (Nowakowski and Czuczman, 2015), subtypes of various cancers identified in the ‘The Cancer Genome Atlas’ (Sanchez-Vega *et al.*, 2018) and patient stratification in allergic diseases (Agache and Akdis, 2019).

Various bioinformatics methods for the computational extraction of subgroups from omics data have been published in recent years. Most of those methods implement novel metrics for the pairwise similarity of samples in a multi-omics setting (Loh *et al.*, 2019; Rappoport and Shamir, 2019; Speicher and Pfeifer, 2015; Wang *et al.*, 2014) followed by standard clustering, or improved clustering approaches addressing the robustness of resulting subgroups (Nguyen *et al.*, 2017).

Here, we present the ‘SGI’ (subgroup identification) package, which implements a new method for the automatic detection of omics-based subgroups. It provides the following novel features compared to previously published methods: (i) the algorithm works hierarchically and can thus identify subgroups of any granularity in complex patient cohorts. (ii) Any sample-wise distance metric can be used for hierarchical clustering, including classical Euclidian distance, or more advanced measures such as similarity network fusion (Wang *et al.*, 2014) and multiple kernel learning (Speicher and Pfeifer, 2015). (iii) It can handle an arbitrary number of clinical variables of interest simultaneously. Combined with the hierarchical approach, this allows the user to explore the complex relationships between various outcomes. (iv) The algorithm is assumption-free and does not require any model fitting, since it only operates on a distance matrix across the omics samples. Moreover, the toolbox implements a comprehensive set of methods to visualize the associations for further interpretation.

## 2 Description

### 2.1 SGI method

**Fig. 1.**
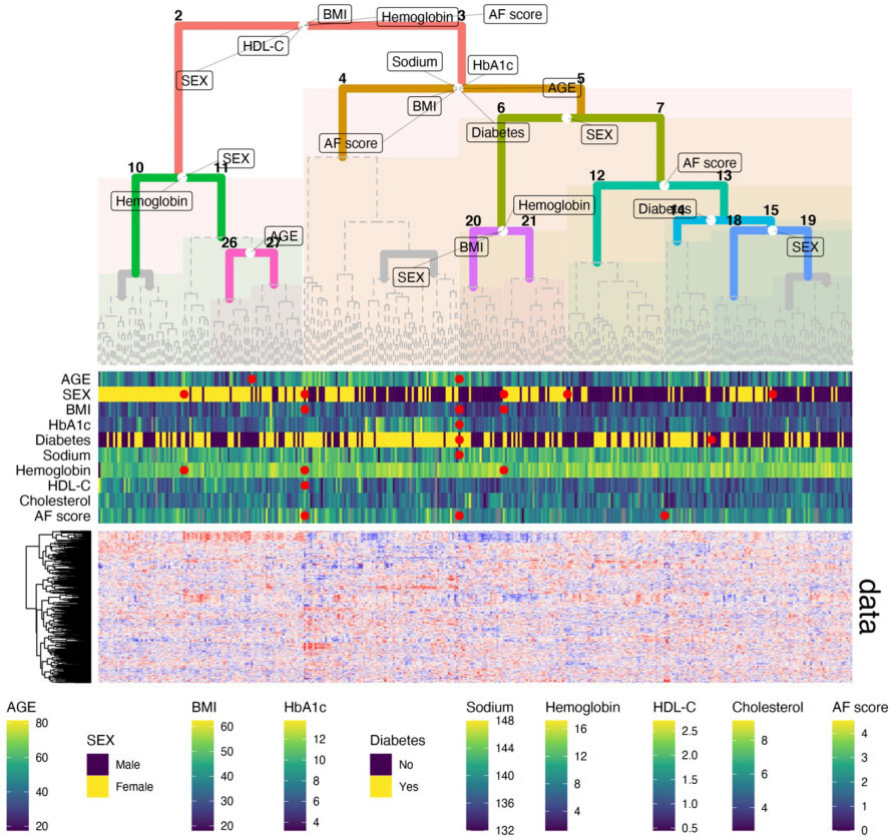
Application example. Blood metabolomics-based clustering of *n* = 356 participants of the QMdiab study. White circles in the tree indicate the left/right splitting points of the samples in the data (note that, these are not centered if the subclusters are of unequal size). Markings on the tree indicate statistically significant associations of the parameter with the respective left and right subgroups at that split. Heatmap track below the tree shows individual values for selected parameters. Red circles between gaps indicate significant results for left versus right at that split and are horizontally aligned with their respective white circles on the tree. Bottom panel shows the metabolomics data matrix behind the clustering

The SGI algorithm generates a hierarchical clustering of samples and runs a two-group association test against the analyzed clinical outcomes at each branching point in the tree. An example output plot is shown in Figure 1, which will be further discussed in the application section below. The algorithm works as follows: (i) **Clustering**. A dendrogram of the samples is generated using standard hierarchical clustering on the input data matrix. The function accepts any hclust object, giving the user full control over the choice of distance and linkage functions. (ii) **Generate valid cluster pairs**. To avoid low-powered calculations in small clusters, the method enumerates all branching points where both left and right subclusters are above a user-defined size threshold. This results in a list of ‘valid’ cluster pairs. SGI runs with a default setting of 5% of the sample size. (iii) **Association analysis.** Run statistical tests with all clinical outcomes for all valid cluster pairs, i.e. compare left versus right subcluster at the respective branching points. SGI has built-in implementations for categorical outcomes (Fisher’s exact tests), continuous outcomes (two-sample *t*-tests) and survival outcomes (log-rank tests). The appropriate test is automatically determined by the toolbox based on the data type of the clinical variable. Furthermore, the user can define arbitrary association functions for more complex data types. (iv) **Multiple testing correction.** Since a dendrogram clusters the samples into cascaded, non-overlapping groups, all statistical tests are strictly independent. Thus, SGI performs Bonferroni multiple testing correction by adjusting each *P*-value by a factor of the number of valid cluster pairs.

In addition to its core functionality, SGI provides support functions to extract, plot, print and summarize all clustering and association results and test statistics, which allows the user to access all intermediate results to further analyze the subgroup results.

### 2.2 Visualization

Association results with multiple outcomes on a hierarchical tree are inherently complex to visualize. The SGI toolbox provides a variety of dedicated functions to visually inspect the statistical associations. This includes tree visualizations of all statistically significant outcome associations that are displayed at the respective branching points and heatmaps of the actual data (Fig. 1). Moreover, the user can generate plots to inspect specific associations, e.g. boxplots of a quantitative clinical outcome between two clusters.

This allows the user to obtain a quick overview of the correlation structure between the input omics dataset, the resulting patient groups and the clinical features that are analyzed. The simultaneous visualization of all data also allows to dissect the relationship between confounding variables, avoiding the predefined choice of a list of confounder variables to correct for.

### 2.3 Multi-omics datasets

The SGI package provides clustering functionality for the analysis of multi-omics datasets, i.e. datasets where more than one omics layer has been measured for the same samples. To this end, SGI generates a joint samples X samples distance matrix from the individual distance matrices of each omics layer. Since different omics layers will have varying numbers of variables, the respective distance values are not at comparable scales. The toolbox thus normalizes each individual distance matrix by its maximum and defines $D=D_{1}/\max(D_{1})+\;D_{2}/\max(D_{2})+\cdots +D_{l}/\max(D_{l})$, with D representing the final distance matrix, $D_{1}$ etc. the original distance matrices and l the number of omics datasets. The approach was adapted from Chavent *et al.* (2018), where it was originally introduced to generate a Ward-like clustering. Notably, the method also works for the normalization of multi-omics contributions for other types of linkages, such as average linkage and complete linkage, and works with any distance metric.

A detailed example of the multi-omics capabilities of SGI on combined plasma, urine and saliva metabolomics data can be found in the example R codes of the github repository.

## 3 Application examples

We will demonstrate the functionality of the SGI package on plasma metabolomics dataset from the ‘QMdiab’ diabetes case/control study with 356 participants (Do *et al.*, 2018; Mook-Kanamori *et al.*, 2014). We chose Euclidean distance with Ward linkage for hierarchical clustering. Outcome parameters were type 2 diabetes diagnosis and nine anthropometric and clinical biochemistry parameters: age, sex, BMI, HbA1c, albumin, hemoglobin, LDL cholesterol, total cholesterol and skin auto-fluorescence (AF score) (Mook-Kanamori *et al.*, 2013). The goal was to determine clusters of study participants defined by their profiles of circulating metabolites, and how these metabolomic clusters correlate with the different clinical parameters. The resulting visualization is shown in Figure 1. The following lines of code directly generate that plot using the SGI package:

# hierarchical clustering

hc = hclust (dist(sgi::qmdiab_plasma),

method = "ward.D2")

# initialize SGI structure

sg = sgi_init(hc, outcomes = sgi::qmdiab_clin)

# run SGI

as = sgi_run(sg)

# generate tree plot, show results for adjusted p-values <=0.05

(gg_tree = plot(as, p_th = 0.05))

# plot overview, including clinical data and metabolomics data matrix

plot_overview (gg_tree = gg_tree, as = as,

outcomes = sgi::qmdiab_clin, xdata = sgi::qmdiab_plasma)

In this R code, sgi::qmdiab_plasma and sgi::qmdiab_clin are data frames holding the metabolomics and clinical variables, respectively. These data frames are contained in the package. The tree shows how metabolomic profiles separate study participants into two major groups at the top level of the tree (clusters 2 versus 3), one with a higher proportion of males, higher BMI, higher HDL-C, as well as higher AF score, and the other group with the reversed effect directions. Inside those two groups, further subgroups were identified; for example, two clusters with different proportions of diabetes, which also associate with Hba1c, age and further diabetes-related risk factors (clusters 4 versus 5).

In addition to the diabetes showcase, we ran SGI on two datasets from The Cancer Genome Atlas, in order to assess whether the algorithm can reproduce known molecular subtypes of cancer. In the first example, we recovered IDH-related mutational subgroups (Ceccarelli *et al.*, 2015; Sanchez-Vega *et al.*, 2018) using SGI on copy number variation data from low-grade glioma samples (Supplementary Material S1). The second example demonstrates how SGI identifies previously described subgroups in uterine corpus endometrial carcinoma samples (Levine *et al*., 2013; Sanchez-Vega *et al.*, 2018), where both our groups and the originally reported groups were based on copy number variation measurements (Supplementary Material S2).

## 4 Conclusion

SGI provides a flexible, unbiased and data-driven way to automatically identify sample subgroups in omics profiles. It identifies and visualizes complex, hierarchical relationships for an arbitrary number of clinical outcomes in a visually intuitive way. The toolbox is easy to use, open source and comes with a series of examples in the online repository.

## Supplementary Material

btab656_Supplementary_Data
