# Supplementary material for: SGI: automatic clinical subgroup identification in omics datasets
Source: Bioinformatics. 2021 Sep 16;38(2):573–6. doi: 10.1093/bioinformatics/btab656 (PMC8723155; doi:10.1093/bioinformatics/btab656)
Supplement: btab656_Supplementary_Data [file btab656_supplementary_data.zip › Supplementary_Material_1_LGG.html]

Supplementary Material 1: Replicating known subtypes of LGG with SGI


# Supplementary Material 1: Replicating known subtypes of LGG with SGI

- 1 Load TCGA LGG CNV and survival data
- 2 Run SGI
- 3 Compare subgroups with known LGG subtypes

In this document, we run SGI on gene-level CNV data from TCGA Low Grade Glioma (LGG) with respect to survival outcomes. CNV and survival data will be downloaded from public repositories, followed by running the SGI algorithm. The identified subgroups will then be compared with known subtypes of LGG. The analysis aims to show that SGI can partially recapitulate known subtypes.

Original publication: https://pubmed.ncbi.nlm.nih.gov/26824661/   
 Subtypes downloaded from: https://pubmed.ncbi.nlm.nih.gov/29625050/

```
library(magrittr)
library(survival)
```

# 1 Load TCGA LGG CNV and survival data

```
# Download files.
# This part has to be executed only once.

dir.create("LGG")

# CNV data
# CNV
download.file(url = "https://tcga-xena-hub.s3.us-east-1.amazonaws.com/download/TCGA.LGG.sampleMap%2FGistic2_CopyNumber_Gistic2_all_data_by_genes.gz",
              destfile = "./LGG/TCGA.LGG.sampleMap_Gistic2_CopyNumber_Gistic2_all_data_by_genes.gz")

# Survival data
download.file(url = "https://tcga-xena-hub.s3.us-east-1.amazonaws.com/download/survival%2FLGG_survival.txt",
              destfile = "./LGG/LGG_survival.txt")
```

Load CNV data

```
cnv =
gzfile('LGG/TCGA.LGG.sampleMap_Gistic2_CopyNumber_Gistic2_all_data_by_genes.gz') %>%
  read.table(header = T, comment.char = "", sep = "\t") %>% {
    mm = as.matrix(t(.[,-1]));
    colnames(mm) = as.character(.[,1]);
    rownames(mm) = colnames(.)[-1];
    mm
  }
# distance matrix
d_cnv = dist(cnv)
```

Load survival data and generate survival outcomes

- **OS:** overall survival
- **DSS:** disease-specific survival
- **DFI:** disease-free interval
- **PFI:** progression-free interval

```
df_surv = read.table(file = "LGG/LGG_survival.txt", header = T, comment.char = "", sep = "\t")
df_surv =
  data.frame(
    # overall survival
    OS = Surv( df_surv$OS.time, df_surv$OS == 1 ),
    # disease specific survival
    DSS = Surv( df_surv$DSS.time, df_surv$DSS == 1 ),
    # disease free survival
    DFI = Surv( df_surv$DFI.time, df_surv$DFI == 1 ),
    # progression free survival
    PFI = Surv( df_surv$PFI.time, df_surv$PFI == 1 ),
    row.names =  make.names(df_surv$sample)
  )

# # patients that both data have available #@mubu, can this be deleted
# inds = intersect(rownames(cnv), rownames(df_surv))
```

# 2 Run SGI

```
library(sgi)
hc = hclust(d_cnv, method = "ward.D2")
sg = sgi_init(hc, length(hc$labels)/20, df_surv[rownames(cnv), ])
as = sgi_run(sg)
ggs = plot_outcomes(sg, as)
```

There is 2 major subgroupings( 3 clusters overall) at the first and second split

```
summary(as)
```

```
## 
## Summary of SGI associations...
## 
## 2vs3 : 2(n=171) vs 3(n=342) at L=2, h=545.16
##  outcome     padj     pval level stat
##       OS 3.74e-04 4.67e-05     2 2.56
##      DSS 2.13e-04 2.67e-05     2 2.81
##      PFI 1.23e-05 1.54e-06     2 2.32
## 
## 4vs5 : 4(n=66) vs 5(n=276) at L=3, h=336.86
##  outcome     padj     pval level   stat
##       OS 4.59e-34 5.74e-35     3 0.0862
##      DSS 1.13e-33 1.41e-34     3 0.0824
##      DFI 6.87e-08 8.58e-09     3 0.0621
##      PFI 2.27e-24 2.83e-25     3  0.166
## 
## 24vs25 : 24(n=31) vs 25(n=159) at L=13, h=116.86
##  outcome   padj    pval level  stat
##      PFI 0.0138 0.00173    13 0.412
```

Inspect Kaplan Meier plots

```
# plot 3 clusters together
# cl. : cluster
sgi_clusters = 
  apply(sgi::get_vcps(sg)[,c("l2","l3")],1,function(x)
    paste0( "cl.", paste(na.omit(rev(x)), collapse = " inside cl.")))

c(
  lapply( c("OS","PFI"), function(i) ggs[[i]][["2vs3"]] + 
            labs(title = i)), 
  lapply( c("OS", "PFI"), function(i) ggs[[i]][["4vs5"]] + 
            labs(title = i) + 
            scale_color_viridis_d() + 
            scale_fill_viridis_d() ),
  lapply( c("OS","PFI"), function(i){
    sfit = survfit(df_surv[rownames(cnv),i]~sgi_clusters)
    ggfortify:::autoplot.survfit(sfit, conf.int.alpha = 0.15) + 
      labs(fill = "sub-group", color = "sub-group")+ 
      theme_minimal() + 
      labs(title = i)
  })
) %>% patchwork::wrap_plots(guides = "collect", ncol = 2)
```

# 3 Compare subgroups with known LGG subtypes

Load data

```
# Download known subtypes, only has to be executed once.
download.file(url = "https://www.cell.com/cms/10.1016/j.cell.2018.03.035/attachment/6622a750-dba1-4498-abfc-1586f9c31515/mmc1.xlsx",
              destfile = "LGG/mmc1.xlsx")

# Load from file
library(readxl)
known_subtypes = as.data.frame( readxl::read_xlsx("LGG/mmc1.xlsx", sheet = 1,skip = 2) )
rownames(known_subtypes)=make.names(known_subtypes$SAMPLE_BARCODE)
known_subtypes = known_subtypes[rownames(cnv),"SUBTYPE",drop = F]
```

Inspect overlap of SGI subgroups and known subtypes

```
table( known_subtypes$SUBTYPE, sgi_clusters) %>% t
```

```
##                   
## sgi_clusters       IDHmut-codel IDHmut-non-codel IDHwt
##   cl.2                      167                2     0
##   cl.4 inside cl.3            0                2    64
##   cl.5 inside cl.3            0              244    28
```

```
# known subtypes annotated on the sgi tree
known_subtypes$SUBTYPE %<>% factor
as0 = sgi_run(sgi_init(hc, length(hc$labels)/20, known_subtypes))
plot_overview(plot(as0), as0, outcomes = known_subtypes, draw_legends = T)
```

Visualize as alluvial plot

```
# prepare plot
library(ggalluvial)
df0 =  reshape2::melt( table( known_subtypes$SUBTYPE, sgi_clusters) )
colnames(df0) = c("known", "sgi", "freq")
df0$sgi = factor(df0$sgi)
```

SGI groups are on the right, and known mutational subtypes of LGG are on the left.

```
# generate plot
ggplot(df0, aes(y = freq, axis1 = known, axis2 = sgi)) +
  geom_alluvium(aes(fill = known), width = 1/12) +
  geom_stratum(width = 1/5.5, aes(fill = after_stat(stratum)), color = "white") +
  geom_text(stat = "stratum", aes(label = after_stat(stratum)), fontface='bold') +
  scale_fill_brewer(type = "qual", palette = "Set1") +
  theme_void() +
  scale_fill_manual(values = c( scales::hue_pal()(2), "#4DAF4A", "#984EA3", "#FF7F00", "#FFFF33")) +
  theme(legend.position = "n")
```

**Conclusion**: There is substantial overlap between sgi subgroups and known mutational subtypes.
