# Supplementary material for: SGI: automatic clinical subgroup identification in omics datasets
Source: Bioinformatics. 2021 Sep 16;38(2):573–6. doi: 10.1093/bioinformatics/btab656 (PMC8723155; doi:10.1093/bioinformatics/btab656)
Supplement: btab656_Supplementary_Data [file btab656_supplementary_data.zip › Supplementary_Material_2_UCEC.html]

Supplementary Material 2: Replicating known subtypes of UCEC with SGI


# Supplementary Material 2: Replicating known subtypes of UCEC with SGI

- 1 Load TCGA UCEC CNV and survival data
- 2 Run SGI
- 3 Compare subgroups with known UCEC subtypes

In this document, we run SGI on gene-level CNV data from TCGA Uterine Corpus Endometrial Carcinoma (UCEC) with respect to survival outcomes. CNV and survival data will be downloaded from public repositories, followed by running the SGI algorithm. The identified subgroups will then be compared with known subtypes of UCEC. The analysis aims to show that SGI can partially recapitulate known subtypes.

Original publication: https://pubmed.ncbi.nlm.nih.gov/23636398/   
 Subtypes downloaded from: https://pubmed.ncbi.nlm.nih.gov/29625050/

```
library(magrittr)
library(survival)
```

# 1 Load TCGA UCEC CNV and survival data

```
# Download files.
# This part has to be executed only once.

dir.create("UCEC")

# CNV data
# CNV
download.file(url = "https://tcga-xena-hub.s3.us-east-1.amazonaws.com/download/TCGA.UCEC.sampleMap%2FGistic2_CopyNumber_Gistic2_all_data_by_genes.gz",
              destfile = "./UCEC/TCGA.UCEC.sampleMap_Gistic2_CopyNumber_Gistic2_all_data_by_genes.gz")

# Survival data
download.file(url = "https://tcga-xena-hub.s3.us-east-1.amazonaws.com/download/survival%2FUCEC_survival.txt",
              destfile = "./UCEC/UCEC_survival.txt")
```

Load CNV data

```
cnv =
gzfile('UCEC/TCGA.UCEC.sampleMap_Gistic2_CopyNumber_Gistic2_all_data_by_genes.gz') %>%
  read.table(header = T, comment.char = "", sep = "\t") %>% {
    mm = as.matrix(t(.[,-1]));
    colnames(mm) = as.character(.[,1]);
    rownames(mm) = colnames(.)[-1];
    mm
  }
# distance matrix
d_cnv = dist(scale(cnv))
```

Load survival data and generate survival outcomes

- **OS:** overall survival
- **DSS:** disease-specific survival
- **DFI:** disease-free interval
- **PFI:** progression-free interval

```
df_surv = read.table(file = "UCEC/UCEC_survival.txt", header = T, comment.char = "", sep = "\t")
df_surv =
  data.frame(
    # overall survival
    OS = Surv( df_surv$OS.time, df_surv$OS == 1 ),
    # disease specific survival
    DSS = Surv( df_surv$DSS.time, df_surv$DSS == 1 ),
    # disease free survival
    DFI = Surv( df_surv$DFI.time, df_surv$DFI == 1 ),
    # progression free survival
    PFI = Surv( df_surv$PFI.time, df_surv$PFI == 1 ),
    row.names =  make.names(df_surv$sample)
  )

# # patients that both data have available #@mubu, can this be deleted
# inds = intersect(rownames(cnv), rownames(df_surv))
```

# 2 Run SGI

```
library(sgi)
hc = hclust(d_cnv, method = "ward.D2")
sg = sgi_init(hc, length(hc$labels)/20, df_surv[rownames(cnv), ])
as = sgi_run(sg)
ggs = plot_outcomes(sg, as)
```

There is one major subgroup at the first split

```
summary(as)
```

```
## 
## Summary of SGI associations...
## 
## 2vs3 : 2(n=359) vs 3(n=180) at L=2, h=1440.16
##  outcome     padj     pval level stat
##       OS 4.59e-07 5.73e-08     2 3.01
##      DSS 7.55e-07 9.44e-08     2 3.76
##      DFI 4.58e-03 5.72e-04     2 2.46
##      PFI 1.07e-08 1.33e-09     2 2.89
## 
## 6vs7 : 6(n=185) vs 7(n=174) at L=4, h=860.73
##  outcome   padj    pval level stat
##      PFI 0.0417 0.00521     4 2.17
```

Inspect Kaplan Meier plots

```
lapply( names(ggs), function(i) ggs[[i]][["2vs3"]] + labs(title = i)) %>%
  patchwork::wrap_plots(guides = "collect")
```

# 3 Compare subgroups with known UCEC subtypes

Load data

```
# Download known subtypes, only has to be executed once.
download.file(url = "https://www.cell.com/cms/10.1016/j.cell.2018.03.035/attachment/6622a750-dba1-4498-abfc-1586f9c31515/mmc1.xlsx",
              destfile = "UCEC/mmc1.xlsx")

# Load from file
library(readxl)
known_subtypes = as.data.frame( readxl::read_xlsx("UCEC/mmc1.xlsx", sheet = 1,skip = 2) )
rownames(known_subtypes)=make.names(known_subtypes$SAMPLE_BARCODE)
known_subtypes = known_subtypes[rownames(cnv),"SUBTYPE",drop = F]
```

Inspect overlap of SGI subgroups and known CN-high subtype

```
table( known_subtypes$SUBTYPE, sgi::get_vcps(sg)[,"l2"])
```

```
##          
##             2   3
##   CN_HIGH  12 151
##   CN_LOW  141   6
##   MSI     141   7
##   POLE     48   1
```

Visualize as alluvial plot

```
# prepare plot
library(ggalluvial)
df0 =  reshape2::melt( table( known_subtypes$SUBTYPE, sgi::get_vcps(sg)[,"l2"]) )
colnames(df0) = c("known", "sgi", "freq")
df0$sgi = factor(df0$sgi, labels = c("2\nbetter OS", "3\nworse OS"))

# generate plot
ggplot(df0, aes(y = freq, axis1 = known, axis2 = sgi)) +
  geom_alluvium(aes(fill = known), width = 1/12) +
  geom_stratum(width = 1/5.5, aes(fill = after_stat(stratum)), color = "white") +
  geom_text(stat = "stratum", aes(label = after_stat(stratum)), fontface='bold') +
  scale_fill_brewer(type = "qual", palette = "Set1") +
  theme_void() +
  scale_fill_manual(values = c( scales::hue_pal()(2), "#4DAF4A", "#984EA3", "#FF7F00", "#FFFF33")) +
  theme(legend.position = "n")
```

**Conclusion**: There is substantial overlap between subgroup 3 (poor survival) and the CN\_HIGH subtype.
